# Antiseptic Dentistry

**Published:** 1894-05

**Authors:** Garrett Newkirk

**Affiliations:** Chicago, Ill.


ARTICLE IV.

### ANTISEPTIC DENTISTRY.

BY GARRETT NEWKIRK, M. D., CHICAGO, ILL.

The subject assigned me by the Executive Committee, for a paper to be read at this meeting, is one of great interest and importance. It is also a question of such magnitude that it cannot be treated exhaustively in a single article of reasonable length.

It shall be my purpose to give briefly some of the reasons why we should, and how we may, apply the principles of antisepsis to every-day office practice.

What do we mean by the term antiseptic?

It is—anti, against—against the septic.

We must learn, then, first, what we mean by the term septic in general, and as applied to dentistry in particular.

In the older dictionaries the term stood for whatever promoted putrefaction. The antiseptic, therefore, was that which should prevent or retard putrefaction. For example, to give the most familiar illustration—moderate heat was septic; extreme heat or cold antiseptic.

The attempt to make fine distinctions between antisepsis and disinfection has caused confusion. In practice, the two are so closely associated they scarcely bear separation, as I think will appear from considerations following:

As I understand it, to use the plainest possible English, sepsis is *poisoning*, by anything which is of the nature of an organic ferment, or the product of such ferment.

Poisoning by any inorganic substance, such as nitric acid or arsenic, or an active principle of vegetable origin, like strychnin or aconite or opium, does not come within the meaning of the term. They are poisons, but not septic; their action is chemical or irritative. They call for antidotes, but are not opposed by antiseptics. Their action is limited by terms of quantity. They may act on certain nerve centers very actively, but if not sufficient to produce paralysis or death, their force is spent; their effects pass. They are not *living* forces; they possess within themselves no multiplying power. They do not increase. True septic agents, on the other hand, do have this power of indefinite, and often very rapid, reproduction and multiplication. They are themselves alive within the living. They are not limited by laws of chemistry, or by rules of quantity, but by *laws of life*—by conditions favorable or unfavorable to reproduction. A septic agent is simply that which contains the germ, the seed, the spore, the reproductive cell of a low form of life, that with conditions favorable to itself is inimical and destructive to that belonging to another and higher form of life.

The act of introduction is *infection*. *Infectious matter is septic matter*. Disinfection is the act of destroying infectious or septic matter.

Antisepsis is to prevent—is against sepsis. It includes necessarily disinfection. The latter is the minor term.

Infection is done variously, by whatever means the septic material is brought into living contact with the body which it poisons. The small-pox patient has probably been infected through the agency of the air, but he may have been inoculated through the skin. The scarlet fever and the measles patients have been infected usually by the road of the lungs, the typhoid fever victim by his alimentary canal.

The milkmaids, whose immunity from small-pox first caught the observation of Jenner, had been infected with vaccina through abrasions on their hands.

By virtue of this wonderful discovery, the people of the civilized world to-day are infected with the same matter introduced at the point of the surgeon's lance.

It is simply voluntary and intelligent infection with one less dangerous virus (an attenuated virus it may be of the same sort) to prevent another infection of deadly fatality.

But mark you, what the surgeon does designedly with the matter of vaccina, he may do unintentionally with the germs of septicemia or the virus of syphilis.

The one condition of infection is this, that the septic poison, the seed, shall be in some manner brought to the fluids of the body—blood, lymph, serum, protoplasm—soil in which it may grow and reproduce its kind.

I was somewhat surprised a year ago to hear a venerable and highly respected member of the dental profession antagonize the modern ideas of disinfection, in this wise—as I remember, he said: "We drink at fountains from cups which have passed from mouth to mouth, and have not been disinfected. We go to hotels and use forks which have been in the mouths of we know not whom; we sit in seats in railroad cars that may be covered with germs; we ride in crowded street cars laden with the breaths of many occupants—we do all this with comparative immunity. Why then should we be so extremely careful beyond ordinary washing of our dental instruments ?"

I say I was surprised because such an argument shows plainly that his thinking had never been thorough enough to go to the core of the subject

The alligator in the Florida swamps is covered with scales for his protection. So is man. The cuticle is no more a part of living tissue than the scales of the alligator. Ordinary agents of attack are repelled and fall harmless from either. A germ to infect must penetrate within this coat of mail. The same is true largely of the mucous membrane. The man is not poisoned because poisons are within his mouth.

Many deadly agents are destroyed by digestion, or passed on without digestion or absorption. To every normally protected surface they are inocuous. If there is no breach in the wall, the enemy besieges in vain. But woe to the surface abraded, the spot unprotected by nature's usual armor. A perfectly whole man might be bathed in the infectious matter of vaccina, there might be a spoonful in his mouth, which could even be swallowed without infection; and yet an amount, so small that it could not be seen, of the same matter introduced on the point of a needle within a living cell would inoculate as certainly as that two and two make four.

The surgeon may open a great abscess; he may perform a laparotomy, where his hands are bathed in septic matter, and no harm follow to him; but if there be anywhere a *broken* surface, even a pin scratch, he is in imminent danger. Woe to him, if with point of infected knife or needle he touches his own blood. His life may pay the forfeit, or disease may scourge him from the crown of his head to the sole of his foot.

The ordinary condition of the cup or glass at the well or fountain is that of smoothness, It is not likely to catch and hold on its edge infectious or other matter. It is frequently washed, and its coolness is unfavorable to the growth of organisms. But if there were on the edge of the cup a ragged point of tin; if that point should by any possibility become infected with the septic germs from the mouth of a drinker, it might inoculate by scratching the lip of another. It is within the range of possibility that such things have taken place.

Forks and spoons are of smooth metal. They are not liable to infection, and they often pass through the best possible condition for disinfection, namely, boiling water. Furthermore, all food well cooked, and all drinks of boiled water, have been disinfected by heat.

As to the argument from the experience of those who ride in crowded street cars and breathe atmospheric "hash," it is probably true that many are infected thereby, so far as infection may be communicated through respiration.

If the integrity of the epithelial covering of the mucous air passages be not perfect, the individual takes his risk of infection by any poison to which he is susceptible, and that is so transmissible. Nevertheless, his risk is infinitely smaller than that of the surgeon who pricks his own skin, or that of his patient, whom he inoculates with the infected instrument.

Let us further illustrate. Here is a person in whose mouth there is a chronic alveolar abscess, or an alveolar ulceration with pyorrhea. Pus is daily discharged and mingled with the fluids of the mouth. More or less for months or years it is mixed with food and drink, and swallowed. It may be at times of most virulent character, yet the individual is not conciously harmed thereby. At the point of disease nature has made a wall, a limit between the living and the dead. The living may pass to the dead, but the dead may not come into the living. The daily swallowed infection matter is digested and destroyed. There may not be absolute immunity, though the danger is comparately slight. But mark you, let a point of steel but touch this infectious matter and then be carried but one inch *in the same mouth*, or to another mouth, with puncture, and there may follow a train of dire results. There may be extensive ulceration, local or general blood poisoning; there may be boils, carbuncles, or pulmonary, hepatic, or abdominal abscess, and not impossibly, death. "*Behold, what great matter a little fire kindleth.*" There was not more comparative potency in Mother O'Leary's lamp and the straw in Mother O'Leary's barn, which burned Chicago, than there is in the microscopic germ of septicemia or syphilis in touch with their fuels.

A young man of eighteen years, brother of one of my patients, applied to a surgeon for a slight operation on his foot and had the misfortune to be inocolated with an infected bistoury. Abscesses followed in the lungs and elsewhere, and after suffering for months, and undergoing several surgical operations, with no end of anxiety on the part of his family, he died.

And all this loss and grief were suffered and borne because a man was ignorant or lazy or careless, and failed to disinfect one little instrument. If he now appreciates the truth, one would think his peace of mind would be gone forever. An experience like this would cloud the sky of a life-time. Where could be the possible compensation or consoling thought to the surgeon who had inoculated a patient with the virus of syphilis? As I understand, it was claimed by the first surgeon in this case that "*Erysipelas had set in.*" We used to hear that expression frequently. Have you noted that it has quite fallen out of the language in these days of antiseptic surgery? It was often merely a term of convenience, to cover unexplainable happenings which we know now were of septic poisoning.

That which holds true of the general surgeon is applicable to the dentist. *He uses a greater number of instruments liable to be infected, in close proximity to territory favorable for infection, than any other man on earth.* Every instrument which enters a carious tooth is likely to be infected by one or more of the agents or products of decay. A smooth excavator may not be, or if it is, may be readily cleansed, but a bur with its many grooves is certain to be, and is not easily cleansed. One of the most, if not *the* most dangerous instrument for infection is the bur when allowed to slip from the cavity and make a punctured wound of the soft parts.

Let me here remind you that of all wounds the puncture is most to be dreaded.

Ordinarily nature protects herself from inoculation by an instantaneous flow of blood, which washes away all foreign matter, or mayhap imprison it within a clot, but the punctured wound defeats her efforts.

The hypodermic syringe is a device to secure absorption by means of a puncture through which nothing may return.

The penetration of the rusty nail into the foot of the boy—the wound by the septic tine of a stable fork, as sources of tetanus, are examples familiar to common experience.

The chance of making a punctured wound with an infected bur, adds another reason for the use of rubber-dam in preparing cavities. How often does the dam ward the bur from contact with soft tissues, or, failing to do this completely, removes the principal part of foreign matter adherent thereto—just as cloth or leather wipes the tooth of the rabid dog or the fang of the rattlesnake, so greatly diminishing the chances of inoculation.

Forcep blades are especially liable to become septic, and remain so, first, because they are used so often on teeth diseased and in mouths diseased; and secondly, because of the roughened surfaces of their jaws. They are especially dangerous because they will be applied to other teeth in other mouths, and crowded down beneath the gums. They would doubtless infect oftener were it not that the parts wounded by them are highly vascular and usually bleed very freely. Nevertheless, given a forcep touched with the germs of pyorrhea, septicemia or syphilis--possibly other poisons of which we are as yet ignorant, how easily might any of these be transmitted from one patient to another.

Of such transmission there are many recorded instances, and little doubt that the unrecorded, if known, would far outnumber those.

In relation to possible infection, rubber-dam clamps may be classed with forceps. Whatever is about the necks of teeth adheres to the clamp, and becoming dry is hard to remove. It may be doubted whether one in fifty of the clamps used by us are kept always surgically clean. As another possible agent, we may not overlook the rubber-dam itself, the edges of which, forced beneath the gums, are sure to carry away some of the adherent secretions of the part, and rubber is particularly hard to disinfect. The passage of rubber-dam from mouth to mouth is one economy certainly that is scarcely "penny wise" while it may be many pounds foolish.

However, as a means of preventing infection by all other means, there is nothing to compare with it or take the place of a piece of clean rubber-dam.

We must remember, too, that intelligent people are thinking of these things, and will continue to think more and more. They observe our methods often more closely than we suppose.

I have purposely omitted so far any mention of the broach and the aseptic management of pulpless teeth, or of teeth the pulps of which are being devitalized, because these special subjects have been of late pretty thoroughly treated and brought to the attention of every intelligent reader of dental literature. I have sought rather to keep in view the surgical principles involved within the whole range of practice, and lead up to certain questions which I wish to ask, and would like everyone to ask himself.

Are we practicing dentistry antiseptically?

There are many, no doubt, who are well informed as to the danger of sepsis, and who realize in theory the necessity of antisepsis, but from lack of a proper system, do not carry out their ideas practically. In order to do this there are required certain special arrangements and conveniences, together with scrupulously careful attention to details. There must be also careful instruction of assistants, and keen oversight that orders are obeyed.

The dentist here may get valuable hints from the general surgeon. How does he go about operating with a view to prevent infection of his patient? First, the surfaces near the part to be operated on are thoroughly cleansed with water and soap, followed by alcohol, and possibly the bichloride solution. The hands of the operator and his assistants are likewise cleansed. Their coats are laid aside, and other clothing covered with clean gowns. The instruments have been made aseptic and are laid on clean napkins. The water to be used has been boiled, and sponges are sterilized. The operation throughout has in view the avoidance of any possible introduction of foreign matter, and the final dressing of the parts is strictly antiseptic.

Should the dentist be any less careful as to surgical cleanliness as to himself and his instruments; and should he not also cleanse the mouths, and especially the teeth, before he proceeds to further operation?

As before said the dentist must have special arrangements and conveniences.

If water pressure be available, the fountain cuspidor. If not, then one nickel-plated, to be cleansed after each patient's use, kept partly filled with water, and *daily scalded.*

Hot water always ready for use on instruments, and for cleansing the hands of the operator. The hands cannot be so thoroughly cleansed with cold water as with warm.

An abundant supply of towels and napkins. There should always be a clean napkin on the bracket on which are laid the instruments, and this should be changed often. It is my habit to buy plain towels, one of which will make two napkins cut to fit the bracket. I take pains to mention this, because I have often observed dentists using merely the cloth cover of the bracket which had become stained and saturated till it was a sight to behold if not to admire. One could scarcely imagine anything better calculated to promote surgical *un*cleanliness.

An indispensable convenience to me for purposes of cleanliness consists in the use of three or four inch squares of bleached muslin, such as one may buy for five or six cents per yard. My assistant keeps a sufficient number of these prepared, and I should scarcely know how to keep house without them.

Am I removing tartar, or treating a case of pyorrhea, one of these squares is always in my left hand, serving to hold the lip, and when the instrument needs wiping it is used for that purpose, and consigned to the waste basket and the fire. I use them for the wiping of burs and excavators, for stripping the soiled cotton from broaches when cleansing pulp canals, for absorbing blood, etc., for laying hold of loose pieces of amalgam or tartar in the mouth, for receiving the tooth just extracted, for wiping the mouth mirror or the hand glass, for use with a little alcohol or chloroform to cleanse the points of the pliers when gummed with sandarac, for removing dirt from the engine hand-piece, etc.

Infinitely better, it seems to me, to use this inexpensive muslin, which may be at once consigned to destruction, than to depend on the heavier, less convenient napkin, that accumulates filth on filth, to be carefully saved for the washtub. It is the rule now, both in surgery and medicine, to dispose of all filth and products of disease by fire rather than the laundry.

A great convenience, one that was suggested to me some years ago by Dr. Harlan, consists in a number of small cups or jars for holding burs. One is able by this means to keep them assorted and only use those which are clean. As a rule, when a bur has been used once or twice its glory has departed, and it should go at once into the waste, or a convenient box kept for those which may be worth resharpening. On the bracket, too, should be a receptacle for burs which have just been used and one may wish to use again after they have been cleaned and disinfected.

All burs when received, either new or resharpened, should be given a coat of some disinfectant oil, for insurance against both rust and infection, and the rule applies equally to excavators, scalers, forceps, clamps, etc., which after cleaning are put in place to await use.

A simple and convenient device for the use of heat to clean instruments is the following: Take a small tin or copper tea-kettle, having a straight spout. Have the tinner attach to the lid of the kettle two or three tubes an inch in diameter and long enough to reach nearly to the bottom. These closed at the bottom and opening outward, may be used for dry heat or oil, while all the long instruments may be dipped in boiling water through the spout.

Any sort of small burner will keep the water at the boiling point with but little trouble or expense.

Antiseptic dentistry, or antiseptic anything, means *cleanliness*. But how can we expect surgical cleanliness if we fail to appreciate the ordinary cleanliness reached by the simplest means. By hot water and soap, the dentist himself, his coat, his hands, and all the belongings of the chair, and bracket may be clean. Then, with a little care surgical cleanliness is added, and antisepsis is complete. I leave the subject at this point, hoping I have developed sufficient interest to cause a free discussion.—*Items of Interest.*

